# Hydrophilic Interaction Chromatography HRMS with Acrylamide Monolithic Columns: A Novel Approach for Intact Antibody Glycoform Characterization

**DOI:** 10.1021/acs.analchem.5c02033

**Published:** 2025-06-16

**Authors:** Annika A. M. van der Zon, LoÏs N. Hana, Huda Husein, Thomas Holmark, Ziran Zhai, Andrea F. G. Gargano

**Affiliations:** † van ‘t Hoff Institute for Molecular Sciences, Analytical Chemistry Group, 1234 University of Amsterdam, Science Park 904, Amsterdam 1098 XH, the Netherlands; ‡ Center of Analytical Sciences Amsterdam, Science Park 904, Amsterdam 1098 XH, the Netherlands

## Abstract

Glycosylation significantly impacts the pharmacokinetics and efficacy of monoclonal antibody (mAb) biotherapeutics. Characterizing mAbs’ glycoform profiles is crucial for optimizing therapeutic outcomes, and intact antibody analysis provides key information about the glycoform combinations present. While state-of-the-art RPLC-MS methods are commonly used for intact mAb analysis, they lack the selectivity to resolve glycoforms and, therefore, may not detect lower-abundance glycoforms. In contrast, HILIC methods have demonstrated good resolving power for middle-up mAb glycoform analysis. However, to date, no application of HILIC has been described to characterize mAb glycoforms at the intact level. This study describes the development of acrylamide monoliths for HILIC-MS intact mAb glycoprofiling. We studied how the porogen composition (octanol and DMSO ratio) in the polymerization mixture affects the column permeability and separation performance. Our findings indicated that increasing the DMSO content increased retention and decreased the peak widths. The optimized HILIC-MS method was applied to analyze five reference intact mAbs (IgG_1_ and IgG_4_). The method demonstrated glycoform selectivity at the intact protein level, achieving baseline separations between single and double Fc glycosylation (e.g., for trastuzumab, resolution (Rs) of 3.62 for G0F vs G0F/G0F) and partial separations between glycoforms differing by one glycan unit (e.g., for trastuzumab, Rs of 1.06 between G0F/G0F and G0F/G1F). Compared to state-of-the-art RPLC-MS, acrylamide-monolith HILIC-MS enabled the measurement of low-abundance glycoforms (e.g., single G0F and M5/M5). The selectivity and sensitivity (ng of sample injection) of this method open opportunities for studies of IgG heterogeneity in bioanalytical applications.

## Graphical abstract

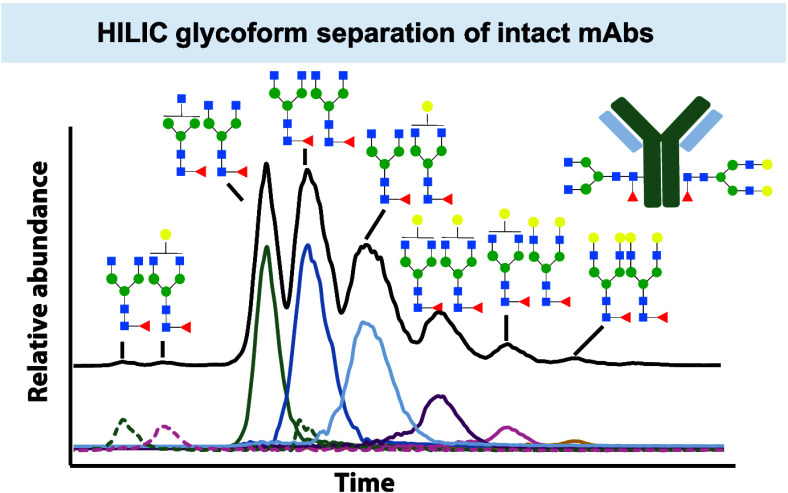

## Introduction

Monoclonal antibodies (mAbs) typically contain N-linked oligosaccharides (N-glycans) attached to the Fc region of the heavy chain. Modifications in their glycosylation profiles can impact their pharmacokinetics and protein efficacy.^1^ Thus, determining the glycoform profile of therapeutic mAbs is essential. Multilevel approaches, including released glycans, peptide, subunit, and intact levels, are therefore used for product characterization.^2,3^ Among these, the characterization of glycoproteins at an intact level provides information about the specific glycoform combinations, some of which cannot be retrieved at other levels (e.g., single N-glycosylation). Moreover, intact protein characterization reduces the sample preparation steps and, therefore, the likelihood of artificial modifications.

However, current separation and mass spectrometry (MS) techniques face limitations in intact-level analysis, hindering glycoform profiling of mAbs, particularly for detailed glycoform characterization. A key challenge lies in effectively separating intact mAb glycoforms.^4^ Reversed-phase liquid chromatography (RPLC) coupled with MS is the most commonly used method for analyzing mAbs.^5,6^ However, RPLC relies on a hydrophobicity-based retention mechanism, which lacks sufficient glycoform selectivity, leading to coelution of the glycoforms and limiting the range of identifiable species. Other approaches, including ion-exchange chromatography (IEC) or capillary electrophoresis (CE), have limited application in resolving neutral glycoforms of mAbs.^7^

Hydrophilic interaction chromatography (HILIC) is a promising alternative for glycoform separations, allowing for more in-depth characterization of glycoproteins by LC-MS approaches. In glycoprotein HILIC separations, neutral stationary phases (amide chemical selectors) are used with mobile-phase gradients of acetonitrile (ACN) to water, in combination with mobile-phase additives, like trifluoroacetic acid (TFA), that decrease the pH of the eluent and allow ion-pair formation with basic protein residues.^8–10^ Under these conditions, glycans significantly contribute to protein retention, enabling high-resolution separation of glycoforms. This makes HILIC an ideal approach for glycoprotein analysis at the intact level,^9,11^ with recent applications reporting high-resolution separations, such as biopharmaceuticals (e.g., erythropoietin),^8,12–15^ and the Fc portion of serum immunoglobulins.^16^

Our group recently described poly­(acrylamide-*co*-N,*N*-methylene-bis­(acrylamide)) monolithic stationary phases for HILIC of proteins.^17^ Monolithic column synthesis conditions can be tuned to modify pore morphology and surface area to create materials specifically designed for macromolecular separations.^18^ Compared with silica-based stationary phases, acrylamide monoliths can be used with reduced percentages of TFA (e.g., 0.005% (v/v)). However, the presence of TFA remains crucial for effective glycoprotein analysis, as demonstrated by Passamonti et al.^19^

We have successfully applied polyacrylamide monoliths to the HILIC-MS characterization of glycoforms from glycoproteins up to 40 kDa, including *N*- and O-glycans from the SARS-CoV-2 spike receptor-binding domain (∼32 kDa), horseradish peroxidase (∼40 kDa), as well as other glycoproteins.^20,21^ This study aims to extend their application to the separation of glycoforms of immunoglobulin G (IgG) mAbs (∼150 kDa). Separating intact mAb glycoforms is challenging due to the combination of slow mass transfer caused by the large size of the antibodies and the small mass difference of the glycans relative to the overall mass of the glycoprotein (e.g., around 1.5% for a single Fc glycan in a mAb of about 150 kDa).^11^ To optimize the separation performance of our monolithic materials, we tested several polymerization conditions to vary their pore properties. This was aimed at increasing the separation performance and reducing potential mAb carryover. A polymerization condition was selected and used for the HILIC-MS analysis of five IgG mAbs. Finally, we compared the masses observed in the analysis of a reference mAb using HILIC-MS with state-of-the-art RPLC-MS analysis.

## Experimental Section

### Chemicals

ACN, methanol, and TFA were purchased from Biosolve B.V. (Valkenswaard, The Netherlands). Hydrochloric acid (37%) was acquired from Acros (Geel, Belgium). Sodium hydroxide, octanol (OctOH), acetone, toluene, dimethyl sulfoxide (DMSO), acrylamide (electrophoresis grade, 99%), *N,N’*-methylenebis­(acrylamide) (99%), 3-(trimethoxysilyl)­propyl methacrylate (98%), 2,2’-azobis­(isobutyronitrile) (AIBN, 99%), myoglobin from equine heart, carbonic anhydrase from bovine heart, bovine serum albumin (BSA), and transferrin from human serum were obtained from Sigma-Aldrich (St. Louis, Missouri, USA). A water Arium 611UV system (Sartorius, Göttingen, Germany) was used to obtain ultrapure water. Trastuzumab (Herceptin), pembrolizumab (Keytruda), ipilimumab (Yervoy), and nivolumab (Opdivo) were received from Amsterdam University Medical Center Pharmacy (Amsterdam, The Netherlands). NISTmAb (reference material 8671, humanized IgG_1K_) was obtained from NIST (Gaithersburg, Maryland, USA). The mAbs were diluted to 50 μg·mL^–1^ in water.

### Preparation of Poly­(acrylamide-*Co*-N,N′-Methylenebis­(Acrylamide)) Monoliths

A bare fused silica capillary (200 μm i.d., 365 μm o.d., 15 cm length) was purchased from Polymicro Technologies (Phoenix, USA). The preparation of the acrylamide monoliths can be divided into two steps: (i) vinylization of the inner surface of the bare fused capillary^22^ (see Section S-1) and (ii) polymerization of the acrylamide-based monoliths. For the polymerization, a polymerization mixture (O26/D74) of 13.70 wt % acrylamide was mixed with 19.43 wt % OctOH. Subsequently, 11.21 wt % *N,N’*-methylenebis (acrylamide) and 55.42 wt % DMSO were added to the mixture. Finally, 0.25 wt % AIBN was added (see Table 1 for other column conditions). Between the steps, the mixture was sonicated for 10 min. The solution was bubbled with nitrogen gas for 1 min. The syringe was first rinsed with DMSO. Then, the polymerization mixture was directly fitted into the column. The ends of the capillary were closed with rubber end plugs. Several capillaries were put into a 500 mL volumetric flask that was filled with ∼40 °C water. The flasks were placed in a water bath (CORIO CD-900F Refrigerated/Heating Circulator, Julabo, USA) at 60 °C for 24 h. After 24 h, 0.5 cm of both capillary ends were cut. Then, the capillaries were flushed with methanol at room temperature at a flow rate of 0.5 μL·min^–1^ for 30 min. The permeability was calculated using Darcy’s law (eq 1). The backpressure of the column was monitored when the flow was adjusted from 0.5 to 4 μL·min^–1^.

**Table 1:** Permeability of Monolithic Stationary Phases Synthesized with Different Ratios of Porogen Composition^a^

	Porogen ratio		
Condition	OctOH (%)	DMSO (%)	Permeability (m^2^); (RSD (%), *n* = 3)
O34/D66	34.0	66.0	5.72·10^–14^ (5)
O31/D69	31.3	68.7	3.67·10^–14^ (7)
O28/D72^b^	28.6	71.4	1.92·10^–14^ (4)
O26/D74	25.9	74.1	7.57·10^–15^ (9)
O23/D77	23.3	76.7	N.A.^c^

a The labeling (e.g., O34/D66) refers to the amount of OctOH and DMSO (wt%) in the polymerization mixture (25 and 49 wt %, respectively). The polymerization mixture was composed of 13.70 wt % acrylamide, 11.21 wt % *N,N*’-methylenebisacrylamide, 0.25 wt % AIBN, and different wt% of OctOH and DMSO. The intrabatch permeability was calculated using eq 1.

b Polymerization mixture described in Passamonti et al. (2021).^17^

c Not assessed due to high back pressure.

According to Darcy’s law, the flow rate (*F*) was multiplied by the dynamic viscosity (η) and the length of the column (*L*) divided by the pressure drop (Δ*P*) and the radius of the column (*r*) to calculate the permeability (*K*_f_).

1 $$K_{f}=\frac{F\cdot \eta \cdot L}{\Delta P\cdot \pi \cdot r^{2}}$$

### HILIC-UV

A Vanquish Neo nano-LC system (Thermo Fisher Scientific, Bremen, Germany) was used. We employed a trap-and-elute configuration to load samples onto the capillary column.^10^ The weak solvent consisted of 2% (v/v) ACN in 98% water. The sample was first loaded onto a C4 trap column (0.3 mm i.d. × 5 mm, 5 μm, 300 Å, Thermo Fisher Scientific, Bremen, Germany) with a flow rate of 10 μL·min^–1^. The mobile phases for the analytical pump were (A) 0.1% (v/v) TFA in 98:2 water:ACN and (B) 0.1% (v/v) TFA in 2:98 water:ACN. The gradient started at 95% B and decreased to 71% B in 3 min. Then, a gradient was programmed from 71 to 66% B in 20 min, followed by a reduction of mobile phase B to 50% in 5 min and subsequently to 20% B in 1 min. After 1 min, there were two washing cycles from 80 to 20% B for each 1 min. A re-equilibration step at 95% B took place for 9.9 min (factor of 2). For all the analyses, the flow rate was set to 1 μL·min^–1^ and carried out at 50 °C. The injection volume was 2 μL (20 μL sample loop). Three blank injections of water were measured between the analyses of different mAb samples. For long-term storage, the columns were stored in 100% B, with both ends of the column inserted into LC vials containing this solvent (Figure S1).

### HILIC-MS

A Q Exactive Plus MS instrument from Thermo Fisher Scientific (Bremen, Germany) was equipped with a Nanospray Flex ion source (Thermo Fisher Scientific, Bremen, Germany). The distance of the emitter from the inlet of the orifice was set to 2 mm. The instrument was operated at 2.00 kV (positive polarity) with a capillary temperature of 300 °C, an S-lens RF level of 100, and an HCD gas pressure of 1. The in-source CID was set to 80 eV for the mAbs. The scan range was set to 600–6,000 *m*/*z* (high mass range (HMR) mode), with 10 microscans and 200 ms as the maximum injection time. The resolution was set to 17,500 (200 *m*/*z*).

### Data Processing

The UV data were processed using Chromeleon 7 (Thermo Fisher Scientific, Bremen, Germany). The raw MS data were visualized in Freestyle. The peak width of the mAbs was determined by using Genesis as the peak detection algorithm, with a peak height of 50%. For the deconvolution of the spectra and calculation of the average mass, Unidec (University of Arizona, Phoenix, USA) was used with the following parameters: sample mass rate of 0.1 Da, picking range of 1 Da, and picking threshold of 0.02.^23^ The raw MS data of intact mAb can be found here: 10.5281/zenodo.15118514.

## Results and Discussion

Acrylamide-based monoliths have proven effective for HILIC separations of proteins and glycoproteins under 40 kDa.^17,19–21^ However, their application to the analysis of intact mAbs remains unexplored. This study addresses this gap by developing and evaluating HILIC monolithic columns specifically designed for intact mAb analysis. To realize this, we (i) optimized the pore structure of acrylamide-based monoliths by systematically varying porogen compositions and assessing their performance in separating model proteins and mAbs using HILIC-UV; (ii) established an optimized method for HILIC-MS analysis with the optimal material and evaluated its performance with five commercially available mAbs (IgG_1_ and IgG_4_); and (iii) compared the glycoform profile for one reference mAb (trastuzumab) obtained by HILIC-MS and RPLC-MS analysis.

### Investigating the Effect of the Porogen Composition on Acrylamide-Based Monolith Chromatographic Performance

Optimizing the pore size distribution of polymer monoliths is crucial for enhancing their separation efficiency, particularly for large molecules, like mAbs, as it directly influences the accessible surface area.^24,25^ Modifying synthesis conditions, such as the amounts and compositions of monomer, cross-linker, initiator, and porogen, will change the morphology of polymer monolithic stationary phases.^26^

The composition of the polymerization mixture used in this study is based on the work of Xie et al.^27^ and Passamonti et al.^17^ Acrylamide was used as the monomer, *N,N*’-methylenebis­(acrylamide) as the cross-linker, and AIBN as the initiator. Their amounts were kept constant. The porogen composition was optimized by systematically varying the ratio of OctOH to DMSO. DMSO served as a “good” solvent, dissolving the monomers, while OctOH, a “poor” solvent, promoted the formation of flow-through pores by inducing earlier polymer precipitation. The DMSO/OctOH ratio influenced the resulting monolith’s pore size distribution, affecting the balance between macropores (>50 nm), mesopores (2–50 nm), and micropores (<2 nm).^28^ While macropores are crucial for flow permeability, mesopores contribute significantly to the material’s surface area, thereby enhancing interactions with the analyte.^26,29^

In this study, we increased the amount of DMSO from 49 to 57 wt % in the polymerization mixture (Table 1). This corresponds to a solvent ratio of OctOH/DMSO from 34/66 to 23/77. We observed a decrease in permeability when increasing DMSO. For example, column conditions O28/D72 and O26/D74 had, respectively, about three and eight times lower permeability with respect to those of O34/D66. O23/D77 presented a too high of a flow resistance and could not be flushed after polymerization. These results follow the expected trends and are expected to occur due to a change in the ratio between macropores and micro- and mesopores (e.g., a higher percentage of micro- and mesopores at a higher percentage of DMSO). The variability in the permeability across different batches was also assessed. The relative standard deviation (RSD) for permeability measurements within each batch (*n* = 3) for all column conditions remained below 9%, indicating good consistency at the intrabatch level. However, when comparing permeability between batches (*n* = 2), some variation was observed (Table S1). Such interbatch differences may be attributed to slight variations in the preparation conditions, which could influence the permeability measurements. Despite this interbatch variability, the overall trend of decreasing permeability with increasing OctOH concentration remained consistent across all batches.

Next, the separation performance of the different monolithic stationary phases was assessed by using a HILIC-UV method with an ACN/water gradient containing 0.1% TFA, and their results were compared in terms of separation quality and carryover. A protein mixture composed of proteins with different molecular weights (myoglobin (17 kDa), carbonic anhydrase (29 kDa), BSA (66 kDa), and transferrin (80 kDa)) was used as a model mixture to assess the separation quality of the materials (Table S2). The four proteins were fully separated with all of the monolithic columns tested (columns O34/D66, O31/D69, O28/D72, and O26/D74, Figure S2). Comparing columns of the same length, the proteins were retained longer with monoliths polymerized with higher DMSO content (O28/D72 and O26/D74). This suggests that under these conditions, a higher amount of micro- and mesopores is generated, increasing the accessible surface area of the material. Limited variation in peak capacity was observed between column conditions (e.g., 30 vs 33 for O34/D66 vs O26/D74, respectively). In terms of peak width, columns with higher OctOH content (O34/D66 and O31/D69) exhibited increased peak widths compared to columns with higher DMSO content (O28/D72 and O26/D74) (e.g., for transferrin, full width at half-maximum (fwhm) of 0.32 min vs 0.25 min for O34/D66 vs O26/D74; full comparison reported in Table S3).

Finally, we compared the different polymerization mixtures in terms of carryover effects. The analysis of intact mAbs requires careful consideration of carryover effects as these large biomolecules are prone to adhering to surfaces and contaminating subsequent analyses.^6^ To assess this, three blanks (injections of water) were measured following the mAb (0.1 μg) measurement. For all the column conditions, the carryover (*n* = 3) after one blank was below 18.5% (based on UV peak area, Figure S3). The carryover after two blanks was about 5%, whereas after three blanks, the carryover was below 2.5%.

We concluded that column conditions O28/D72 and O26/D74 had similar results and demonstrated the best consistency within a column batch, narrower peak widths, and the lowest carryover. Given the slight improvement in separation performance obtained with the O26/D74, we tested this column for intact mAb separations.

### Separation of Glycoforms of Mabs at the Intact Level

ACN/water mobile phases with TFA (e.g., 0.1% (v/v)) are commonly used in HILIC, as demonstrated in several studies,^3,19,30–32^ and are therefore applied in our study. While steep gradients (e.g., 90 to 50% ACN in 20 min) are used to separate mixtures of proteins with large differences in terms of amino acid sequence composition,^10^ shallow gradients (e.g., 5–10% reduction of ACN over 30 min) are necessary to achieve highly efficient glycoform separations.^33^

Therefore, we began our investigation by studying the effect of reducing the ACN gradient window from 20% (85–65% (v/v) ACN) to 5% (71–66% (v/v) ACN) over 20 min (Figure 1). Trastuzumab was used as a model mAb for these experiments. Reducing the steepness of the mobile phase gradient resulted in a substantial improvement in glycoform resolution (Rs) (e.g., Rs between G0F/G0F and G0F/G1F of 0.45, 0.79, and 1.06 at 1%, 0.5%, and 0.2% B/min, respectively). This enabled the separation of up to six glycoform peaks at 0.2% B/min compared to two glycoform peaks at 1% B/min (Figure 1). HILIC-UV separations under these conditions were attempted, resulting in lower separation performance and only partial separations (Figure S5). We suggest that the separation loss is related to the large sample mass injection needed to obtain clear UV signals (0.5 μg using UV vs 0.1 μg in MS) and system dead volumes. The optimized conditions were tested with both O28/D72 and O26/D74 columns, confirming the observation from the analysis of the protein mixture, with O26/D74 offering better separation performance compared to O28/D72 (Figure S6).

**Figure 1:**
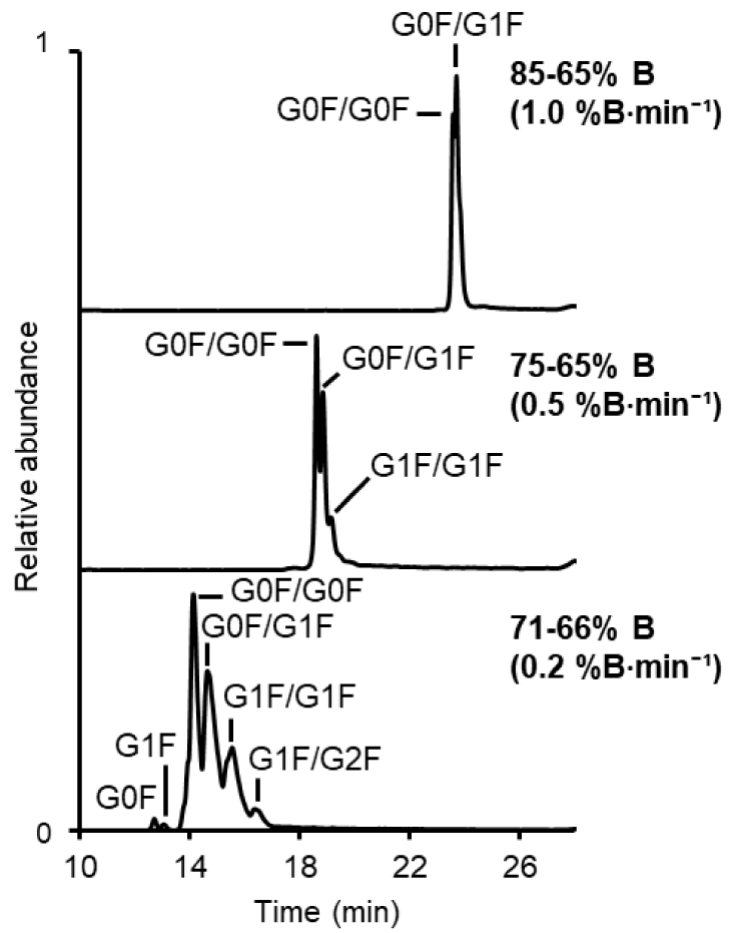
BPC of trastuzumab (0.1 mg·mL^–1^, 1 μL injection volume) showing the influence of the steepness of the ACN gradient on the glycoform separation. Top to bottom: gradient from 85 to 65% B (steepness 1.0%B·min^–1^), 75–65% B (steepness 0.5%B·min^–1^), and 71–66% B (steepness 0.2%B·min^–1^) in 20 min. Details of the glycans are given in Table S4. Isomeric structures such as G0F/G2F are also possible but not illustrated.

The optimized method conditions were used for the HILIC-MS analysis of five IgG mAbs, each injected at 100 ng per analysis: three IgG_1_ mAbs (trastuzumab, NISTmAb, and ipilimumab) and two IgG_4_ mAbs (nivolumab and pembrolizumab). All analyzed mAbs are Fc glycosylated and have high similarity in terms of Fc amino acid (AA) sequence (with respect to trastuzumab % identity of 100% for NISTmAb, missing one AA; ≈99% for ipilimumab, with two AAs different; and ≈95% for the IgG_4_ models, with nine AAs different) but show significant differences in their Fab subunit (% identity between ≈85% and 76%, with up to 60 AA differences). Details of the structural characteristics of the analyzed model mAbs are reported in Section S4.

The base peak chromatograms (BPCs) and extracted ion chromatograms (EICs) from their LC-MS analysis are shown in Figures 2 and S7, and the glycoform structures are summarized in Table S4. The five mAbs eluted between 11 and 17 min. For the same glycoforms (e.g., G0F/G0F) across different mAbs, retention times varied within a 1- to 2-min window (e.g., around 12 min for single Fc glycosylation G0F). Peak widths differed between mAbs (e.g., narrower G0F/G1F peaks for NISTmAb compared to pembrolizumab, Table 2) and were generally broader for larger glycans. These differences did not appear to correlate with properties related to AA sequences, such as isoelectric point, sequence composition, or molecular weight (Table S5). Multiple distributions were observed in the EIC and were more pronounced in the case of larger glycans (e.g., G1F/G2F, Figure S7), suggesting the presence and separation of isomeric structures. While definitive identification of these species remains challenging, we propose several potential explanations: these could include deamidation variants (which HILIC can resolve at the peptide level),^34^ glycated species (as suggested in Figure S8), resolution of isomeric glycans (e.g., G1F isomers observed at the released glycan level),^35^ or even disulfide bond scrambling.

**Table 2:** Comparison of Separation Performances (Number of Separated EIC Glycoform Peaks, Retention Time, Peak Width at 50% Height (Width), and Resolution (Rs)) in HILIC MS Analysis of Five mAbs (Trastuzumab, NISTmAb, Ipilimumab, Nivolumab, and Pembrolizumab)^a^

		**G0F**		G0F/G0F			G0F/G1F		
mAb	Peaks^b^	Time^c^ (min)	Width (min)	Time^c^ (min)	Width (min)	Rs^d^	Time^c^ (min)	Width (min)	Rs^e^
Trastuzumab (IgG_1_)	6	12.85	0.20	14.23	0.25	3.62	14.76	0.34	1.06
NISTmAb (IgG_1_)	6	12.24	0.21	14.03	0.21	5.03	14.46	0.26	1.08
Ipilimumab (IgG_1_)	3	11.70	0.21	12.99	0.21	3.62	13.80	0.26	2.03
Nivolumab (IgG_4_)	5	12.19	0.20	13.95	0.21	5.07	14.78	0.49	1.40
Pembrolizumab (IgG_4_)	4	11.59	0.15	12.38	0.17	2.91	12.67	0.38	0.62

a More information on the mAbs properties are reported in Section S4.

b EIC peaks with Rs > 0.8.

c 5.77 min is subtracted from the retention time, corresponding to the time needed for the RPLC trap loading and elution to the HILIC column.

d Calculated between G0F and G0F/G0F.

e Calculated between G0F/G0F and G0F/G1F.

**Figure 2:**
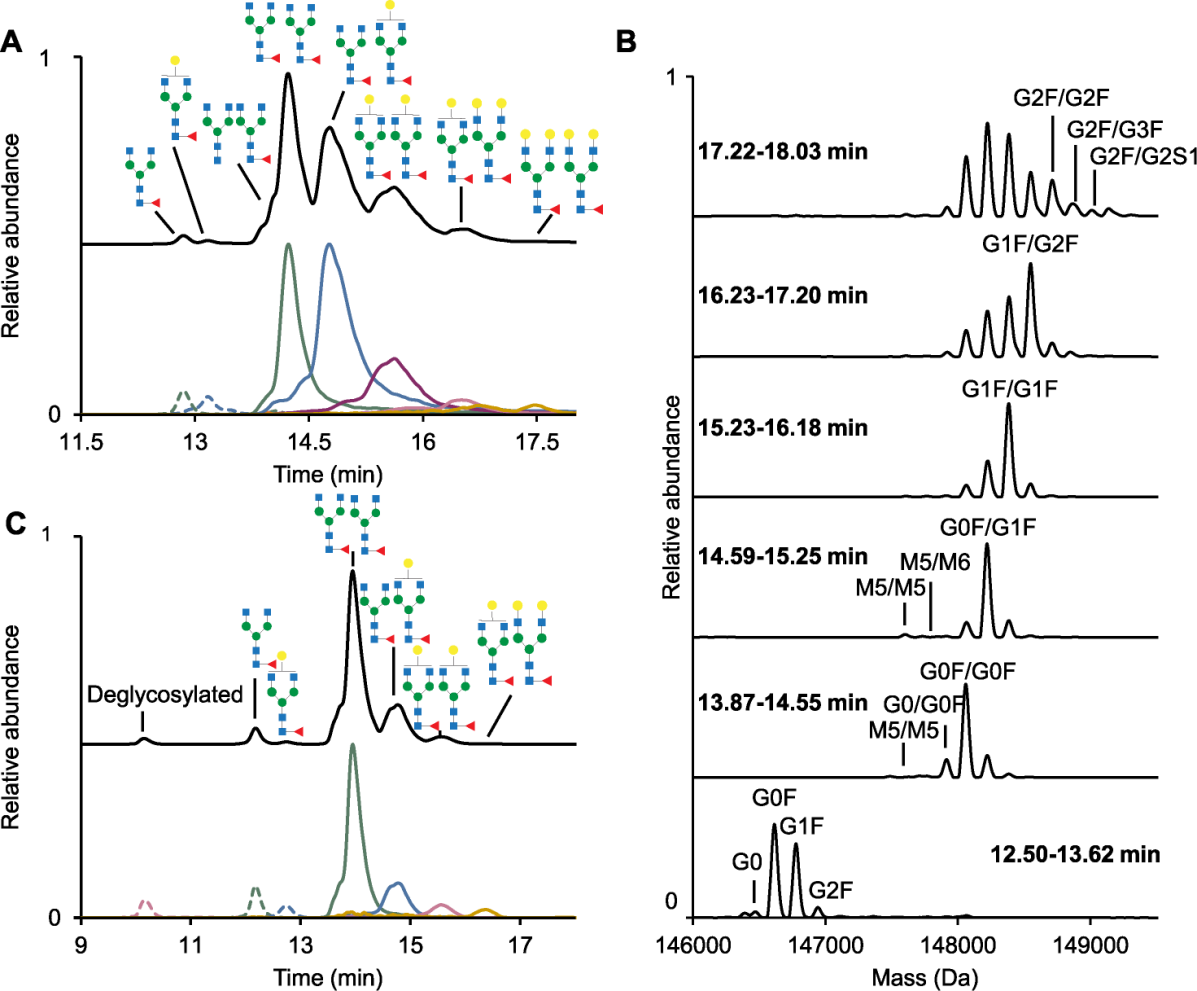
(A) BPC with EIC traces of separated glycoforms of trastuzumab, (B) Deconvoluted spectra of trastuzumab at different elution times, expressed in min, as shown in each panel, (C) BPC with EIC traces of separated glycoforms of nivolumab. The EICs of single G0F, G1F, and deglycosylated peaks (dotted) are five times scaled. Details on the glycans and EIC traces are described in Tables S4 and S7. The glycoforms are illustrated according to ref 38. Isomeric structures such as G0F and G2F are also possible but not illustrated.

Glycoform separations were observed in all BPCs of both IgG_1_ and IgG_4_ mAbs, with three to six glycoform peaks (EIC Rs > 0.8) observed depending on the mAb (Figure S7 and Table 2). The BPC profiles generally reflected the abundance of the different glycoforms present. NISTmAb and trastuzumab showed four main glycoforms (90% of the observed signal, G0F/G0F to G1F/G2F), and nivolumab and ipilimumab showed two main glycoforms (95% of the signal, G0F/G0F and G0F/G1F). Similar to what was described in the HILIC analysis of Fc subunits of mAbs,^3^ the elution order of the Fc glycoforms is related to the mass of the glycan (e.g., G0F/G1F elutes after G0F/G0F). mAb glycoforms with a single Fc glycan (the most abundant being G0F and G1F) were baseline separated from the main isoform having two Fc glycosylations (e.g., G0F vs G0F/G0F, Rs between 2.91 and 5.07, Table 2 and Figure S7). The resolution between two glycoforms was lower (e.g., G0F/G0F and G0F/G1F from 0.62 to 2.03). In addition to nivolumab, we observed a low-abundance deglycosylated peak, baseline resolved from the single Fc-glycosylated peak (Figure S7).

We attempted to replicate similar separations using a commercially available analytical-scale silica-based HILIC column (150 × 2.1 mm I.D., 1.7 μm, 300 Å glycoprotein amide; details and conditions summarized in Table S8) previously applied for glycoprotein analysis.^36,37^ Using the same mobile phase and shallow gradients (2% B in 20 min), we observed coelution of the glycoforms of trastuzumab (Figure S9). We suggest that this difference between silica and acrylamide monolithic columns may be related to the material’s chemistry and/or morphology. Specifically, the silica column may have residual silanols, which act as acidic interaction sites, leading to secondary ion-exchange interactions with the mAb, which may negatively influence the separation of the glycoforms. Furthermore, the particle-bed morphology of the silica column, compared to the monolithic structure of our columns, may also contribute to the observed differences.

To investigate this further, we performed HILIC-UV experiments with both silica-based and acrylamide monolithic columns, varying the TFA concentration from 0.02 to 0.3% (v/v) and comparing trastuzumab elution under similar conditions (programmed gradient time/t0) (Figure S10). The silica column showed no glycoform separation at 0.3% (v/v) TFA, while partial separation was observed with the monolithic column (confirmed by HILIC-MS experiments in the case of 0.1% (v/v) TFA). At 0.02% (v/v) TFA, recoveries were low for both columns, indicating the importance of the concentration of TFA in the separation. In the case of acrylamide monoliths, the low recovery could also be related to interaction with silica (also present in the C4 trap column used for injection in HILIC experiments). Moreover, with the silica column, elution time remained essentially unchanged between 0.1% (v/v) and 0.02% (v/v) TFA, but the peak width significantly broadened, suggesting the presence of secondary ion-exchange interactions. Although more pronounced tailing was observed for the monolithic column at 0.02% (v/v) TFA, this column exhibited a consistent reduction in retention time as the TFA concentration decreased from 0.3 to 0.02% (v/v).

### Characterization of Low-Abundance Glycoforms Using HILIC-MS Compared to RPLC-MS

We then compared trastuzumab analysis using our HILIC-MS method with state-of-the-art RPLC-MS (conditions in Table S9).^39^ RPLC-MS resulted in the coelution of glycoforms into a single peak, allowing for identification of the five most abundant glycoforms (Figure S11, Tables 3 and S9). In contrast, HILIC-MS chromatographically resolved different glycoforms, enabling the observation and deconvolution of low-abundance species such as single Fc glycosylation (e.g., G0F) or high-mannose glycans (e.g., M5/M5) (Figure 2B). HILIC-MS identified a total of 16 unique glycoforms (Tables 3 and S10). While other RPLC-MS studies (e.g., Zhu et al.^40^ have tentatively assigned a greater number of glycoforms, these assignments often rely on coeluting peaks, which in some cases are poorly mass resolved. In contrast, our HILIC-MS results enable the assignment of most glycoforms based on clearly distinguished, time-resolved masses. Furthermore, to the best of our knowledge, no RPLC-MS study has reported the observation of single N-glycans.^40^

**Table 3:** Relative Abundance (%) of Glycoform Combinations of Trastuzumab Measured with HILIC MS and RPLC MS (Table S10)­^a^

Glycoforms	Theoretical mass (Da)	HILIC (%)	RPLC (%)
G0	146,465.7	0.2	N.A.
G0F	146,611.9	0.9	N.A.
G1F	146,774.0	0.6	N.A.
G2F	146,936.2	0.2	N.A.
M5/M5	147,600.7	0.9	N.A.
M5/M6	147,762.9	0.9	N.A.
G0/G0	147,764.9	0.9	N.A.
G0/G0F	147,911.1	6.3	N.A.
G0F/G0F	148,057.2	29.5	26.2
G0F/G1F	148,219.3	31.5	35.6
G1F/G1F or G0F/G2F	148,381.5	20.0	25.2
G1F/G2F	148,543.7	6.1	9.5
G2F/G2F	148,705.8	1.2	3.6
G2F/G3F or G2F/G2F + 1 mannose (glycation)	148,867.8	0.5	N.A.
G2F/G2S1	148,997.1	0.2	N.A.

a Details of the glycans are described in Table S4. A maximum mass error of 10 Da was taken into account (Table S10). N.A. means “not assessed”; these glycoforms could not be identified. Conditions of RPLC-MS analysis are described in Table S9.

Finally, enzymatic deglycosylation of trastuzumab (using PNGase F), followed by HILIC-MS analysis (Figure S8) revealed the presence of a minor percentage (below 1%) of hexose-glycated species, which were partially separated by HILIC. Glycation, a nonenzymatic modification of lysine residues or N-terminal amines with monosaccharides, has been described in mAbs. It results in the introduction of isomeric species of glycoforms as hexose sugars add 162 Da, the same mass increment as galactose.^41^ We suggest that the partial separation of these species is observed in the analysis of the nondeglycosylated mAb, contributing to extra peaks in the EIC. Glycated variants are not separated and, therefore, observed with RPLC and can only be observed upon deglycosylation.

## Conclusion

In conclusion, this study demonstrates the significant potential of acrylamide monolithic stationary phases for the high-resolution separation of mAb glycoforms. Optimization of the porogen composition, specifically increasing DMSO content, enhanced the stationary phase’s surface area and selectivity while reducing peak widths. The optimized acrylamide monolithic columns enabled, for the first time, detailed glycoform separations of intact mAbs (IgG_1_ and IgG_4_) using HILIC-MS. This approach offers a distinct advantage over conventional RPLC-MS, providing more comprehensive glycoform profiling and enabling the characterization of low-abundance species. The sensitivity of the method, thanks to the capillary column format, allows for reduced sample injection amounts (100 ng), making it promising for IgG studies in sample-limited bioanalytical applications.

## Supplementary Material



## Notes

Additional experimental details and data, including porogen ratio optimization, separation of intact glycoforms of the five mAbs, identification of trastuzumab glycoforms, and comparison with commercial amide column and RPLC-MS (PDF)
