# Supplementary material for: Hydrophilic Interaction Chromatography HRMS with Acrylamide Monolithic Columns: A Novel Approach for Intact Antibody Glycoform Characterization
Source: Anal Chem. 2025 Jun 16;97(25):13569–76. doi: 10.1021/acs.analchem.5c02033 (PMC12224157; doi:10.1021/acs.analchem.5c02033)
Supplement: Supplementary file 1 [file ac5c02033_si_001.pdf]

Supporting Information

Hydrophilic Interaction Chromatography HRMS with Acrylamide Monolithic Columns: A Novel Approach for Intact Antibody Glycoform Characterization

Annika A.M. van der Zon[1,2,*], Loïs N. Hana[1,2], Huda Husein[1,2], Thomas Holmark[1,2], Ziran Zhai[1,2], and Andrea F.G. Gargano[1,2,*]

[1] University of Amsterdam, van 't Hoff Institute for Molecular Sciences, Analytical Chemistry Group, Science Park 904, 1098 XH Amsterdam, The Netherlands

[2] Center of Analytical Sciences Amsterdam, Science Park 904, 1098 XH Amsterdam, The Netherlands

[*] Correspondence: a.a.m.vanderzon@uva.nl and a.gargano@uva.nl

Contents

S-1 Preparation of acrylamide monoliths ____ S3
S-2 Performance monolithic columns ____ S4
S-3 Nomenclature of glycoforms ____ S7
S-4 mAbs properties and sequences ____ S9
S-5 Separation of glycoforms of mAbs at intact level ____ S12
S-6 Comparison with RPLC-MS ____ S18
References ____ S20

S-1 Preparation of acrylamide monoliths

Methodology of vinylization of the inner surface of bare fused silica capillary: This protocol was adopted from [1]. Shortly summarized, the capillary column was rinsed with acetone and water. Then, the capillary column was flushed with 0.2 M NaOH until a basic pH was detected at the open end of the capillary. The pH was checked by using pH paper. The capillary was installed on a syringe pump at a flow rate of 15 $\mu L \cdot hr^{-1}$ for 30 min. Afterwards, the capillary was rinsed with water until a neutral pH was detected, followed by flushing the capillary with 0.2 M HCl until an acidic pH was detected. The capillary was flushed again at 15 $\mu L \cdot hr^{-1}$ for 30 min. Subsequently, the capillary was rinsed with water until a neutral pH was detected, followed by rinsing with acetone and toluene. After this, the capillary was filled with the derivatization reagent composed of a 20 wt% solution of 3-trymethoxysilyl-propyl methacrylate in toluene at 15 $\mu L \cdot hr^{-1}$ for 120 min. After 2 hr, the capillary was washed with toluene and blown dry with pressurized nitrogen (2 min). The condensation reaction of the silanol groups took place overnight at room temperature.

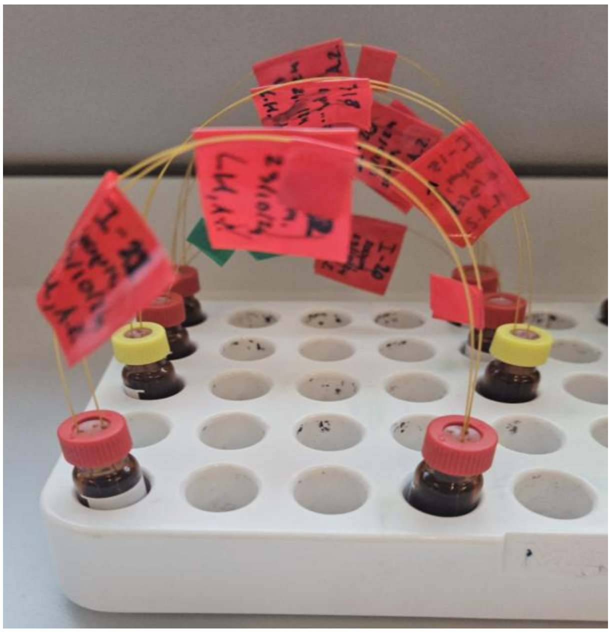

Figure S1: Both ends of the monolithic columns were stored in 0.1% (v/v) TFA in 98% ACN (mobile phase B).

Table S1: Permeability values of columns synthesized under different column conditions between two batches (interbatch). The permeability was calculated by using Equation 1. The relative standard deviation (RSD) is listed.

Column condition	Permeability (m^2)	RSD (%)
O34/D66	$6.0 \cdot 10^{-14}$	3.2
O31/D69	$4.9 \cdot 10^{-14}$	8.0
O28/D72	$2.0 \cdot 10^{-14}$	3.7
O26/D74	$6.4 \cdot 10^{-15}$	9.4
O23/D77	N.A.*	N.A.*

*N.A.= not assessed

S-2 Performance monolithic columns

Table S2: Gradient condition for the separation of the protein mix (myoglobin, carbonic anhydrase, BSA, and transferrin). This method was operated on an UltiMate RSLC3000 nano-LC system (Thermo Fisher Scientific, Bremen, Germany). Additional information is addressed in Experimental section.

Mobile phase A	0.1% (v/v) TFA in 98% water + 2% ACN
Mobile phase B	0.1% (v/v) TFA in 98% ACN + 2% water
Loading solvent	2% ACN in 98% water
Trap	C4 trap column (0.3 mm i.d. x 5 mm, 5 µm, 300 Å) (Thermo Fisher Scientific, Bremen, Germany)
Flow	1.00 µL·min^{-1}
Column temperature	50 °C
Injection volume	1 µL (100 ng)
Detection	214 and 280 nm
Time (min)	Mobile phase B (%)
0.000	94.0
4.000	75.0
5.875	60.0
5.900	60.0
14.230	45.0
16.000	20.0
18.000	20.0
18.100	94.0
25.000	94.0

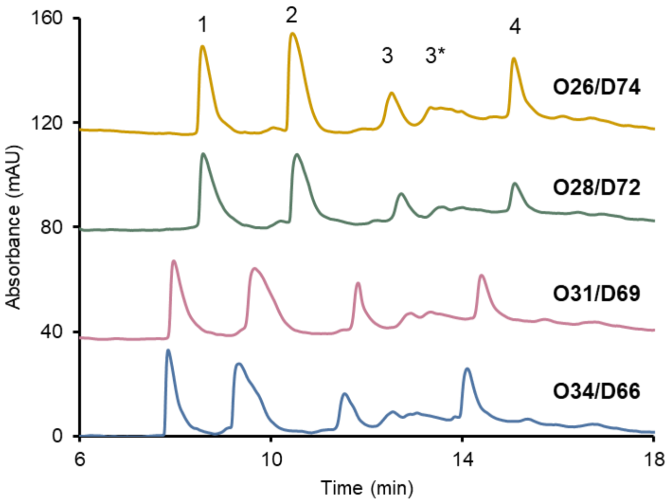

Figure S2: HILIC separation of protein mix (myoglobin (1), carbonic anhydrase (2), BSA (3), and transferrin (4)) measured under four different column conditions; O34/D66 (blue), O31/D69 (pink), O28/D72 (green), and O26/D74 (yellow). Peak 3* are the oligomers of BSA. Additional method conditions are described in Table S2. The dimensions of the columns were 200 µm i.d. x 15 cm length.

Table S3: Integration of parameters (retention time, peak width at half height (FWHM)), and peak capacity (n_c)) based on protein mix measured with four different column conditions. The peak capacity is calculated using Eq. S1. See Table S2 for detailed LC settings.

	Myoglobin		Carbonic Anhydrase		BSA		Transferrin		Protein Mixture
**Column**	**Time (min); (RSD (%))**	**FWHM(min); (RSD (%))**	**Time (min); (RSD (%))**	**FWHM(min); (RSD (%))**	**Time(min); (RSD (%))**	**FWHM(min); (RSD (%))**	**Time (min); (RSD (%))**	**FWHM(min); (RSD (%))**	**n_c; (RSD (%))**
O34/D66	7.93; (1)	0.24; (12)	9.42; (1)	0.66; (12)	11.60; (2)	0.36; (7)	14.08; (1)	0.32; (10)	29.97; (11)
O31/D69	7.95; (1)	0.26; (18)	9.58; (3)	0.53; (10)	11.86; (2)	0.32; (32)	14.38; (2)	0.30; (22)	25.80; (1)
O28/D72	8.30; (3)	0.29; (28)	10.03; (4)	0.40; (10)	12.20; (4)	0.32; (9)	14.70; (3)	0.26; (17)	30.91; (12)
O26/D74	8.38; (4)	0.28; (23)	10.14; (6)	0.35; (7)	12.23; (5)	0.29; (5)	14.77; (4)	0.25; (7)	33.26; (2)

Eq.S1: Peak capacity (n_c), where the gradient time (t_g) and the peak width at half height (FWHM) are taken into account.

$$n_c = \frac{t_g}{1.7\ x\ FWHM} + 1$$

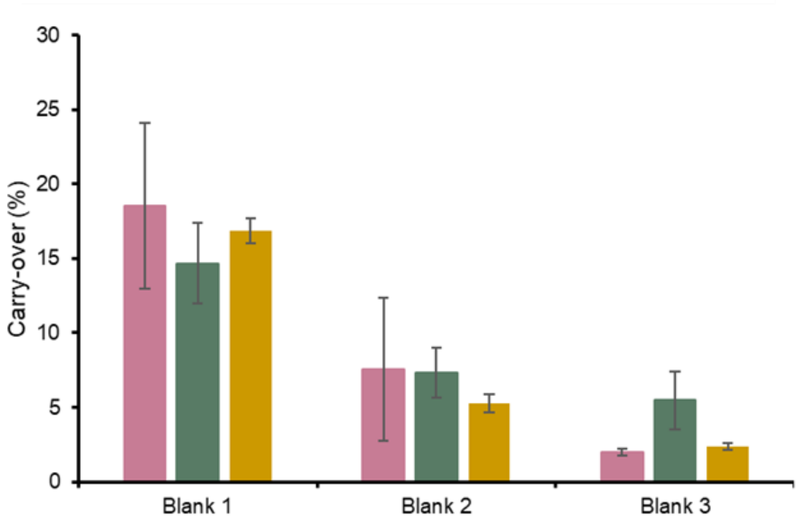

Figure S3: Carry-over after the injection of 100 ng trastuzumab after measuring three blanks (water) using three different columns; O31/D69 (pink), O28/D72 (green), and O26/D74 (yellow). The following LC conditions were performed: 100 ng mAb was injected into the column. The gradient started at 94% B and ramped down to 60% B in 9 min and kept it for 1 min, in 7 min it went to 10% B for 8 min, and went back to 94% B in 0.1 min. The re-equilibration time was 5 min.

S-3 Nomenclature of glycoforms

Table S4: Nomenclature and symbolic representations of glycoforms with their theoretical masses. The glycoforms are illustrated according to [2].

Structure	Name*	Short name	Theoretical mass (Da)
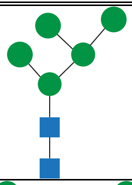	H5N2	M5	1217.09
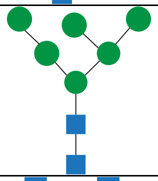	H6N2	M6	1379.23
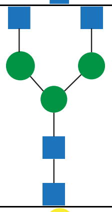	H3N4	G0	1299.19
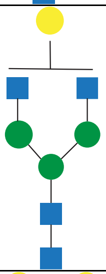	H4N4	G1	1461.33
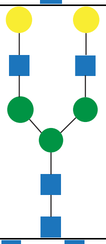	H5N4	G2	1623.48
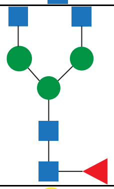	H3N4F1	G0F	1445.34
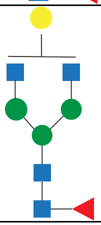	H4N4F1	G1F	1607.48

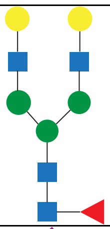	H5N4F1	G2F	1769.62
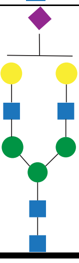	H5N4S1	G2S1	1914.73

*Glycan residues: hexose (H, (mannose, green circle) or (galactose, yellow circle)), N-acetylglucosamine (N, blue square), fucose (F, red triangle). and N-acetylneuraminic acid (S, purple diamond shape).

S-4 mAbs properties and sequences

Table S5: Information regarding IgG subclass, molecular weight, and isoelectric point (pI) of the measured mAbs (trastuzumab, NISTmAb, ipilimumab, pembrolizumab, and nivolumab) in this study.

	**IgG class**	**Molecular weight[I]**	**pI theoretical[II]**	**pI cIEF[III] main peak**
Trastuzumab	IgG_1	145,166.5 Da	8.8-9.1	9.1
NISTmAb	IgG_1	145,147.1 Da	8.8-9.2	9.1[III]
Ipilimumab	IgG_1	145,100.4 Da	8.9-9.2	9.2
Pembrolizumab	IgG_4	145,997.3 Da	7.4-7.8	7.6
Nivolumab	IgG_4	143,330.1 Da	8.0-8.3	8.0

[I] For the molecular weight, N-terminal pyroglutamate conversion, loss of C-terminal lysine clipping, and disulfide bondings are taken into account.; [II] The pIs are extracted from Goyon *et al.* [3]; [III] The pI measured with cIEF is taken from Turner and Schiel. [4]

A) Fc Sequence Blast Summary (https://blast.ncbi.nlm.nih.gov/Blast.cgi)

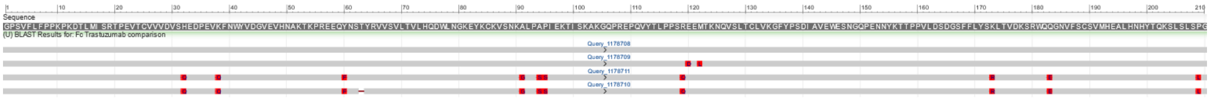

select all 4 sequences selected — Graphics | Distance tree of results | Multiple alignment | MSA Viewer

Description	Scientific Name	Max Score	Total Score	Query Cover	E value	Per. Ident	Acc. Len	Accession
NISTmAb		436	436	100%	2e-162	100.00%	211	Query_1178708
Ipilimumab		433	433	100%	2e-161	99.05%	211	Query_1178709
Pembrolizumab		418	418	100%	2e-155	95.24%	211	Query_1178711
Nivolumab		411	411	100%	5e-153	94.76%	210	Query_1178710

B) Fab Sequence Blast Summary (https://blast.ncbi.nlm.nih.gov/Blast.cgi)

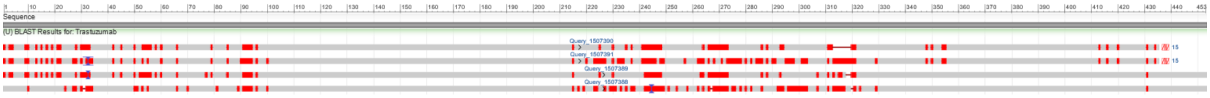

Description	Scientific Name	Max Score	Total Score	Query Cover	E value	Per. Ident	Acc. Len	Accession
Ipilimumab		746	746	100%	0.0	84.80%	452	Query_1507389
Nivolumab		689	689	96%	0.0	81.84%	443	Query_1507390
NIST		686	686	100%	0.0	80.66%	452	Query_1507388
Pembroluzimab		639	639	96%	0.0	76.08%	454	Query_1507391

Figure S4: Blast differences in the **(A)** Fc and **(B)** Fab chain subunit amino acid composition between the five model mAb studied**.** The red dots indicate the amino acid differences

Amino acid sequence (Fc/2 subunit) of measured mAbs

Difference in the Fc/2 subunit (after in silico IdeS release from heavy chain) amino acid composition between five of the studied mAb. In pink, the glycosylation site is indicated, and differences in the amino acid sequence are marked in blue and green.

Fc sequences (from IdeS cleavage)

- **Fc trastuzumab reference**

GPSVFLFPPKPKDTLMISRTPEVTCVVVDVSHEDPEVKFNWYVDGVEVHNAKTKPREEQYNSTYRVV
SVLTVLHQDWLNGKEYKCKVSNKALPAPIEKTISKAKGQPREPQVYTLPPSREEMTKNQVSLTC
LVKGFYPSDIAVEWESNGQPENNYKTTPPVLDSDGSFFLYSKLTVDKSRWQQGNVFSCSV
MHEALHNHYTQKSLSLSPG

- **Fc NISTmAb 100% identity (one additional K)**

GPSVFLFPPKPKDTLMISRTPEVTCVVVDVSHEDPEVKFNWYVDGVEVHNAKTKPREEQYNSTYRVV
SVLTVLHQDWLNGKEYKCKVSNKALPAPIEKTISKAKGQPREPQVYTLPPSREEMTKNQVSLTCLVKG
FYPSDIAVEWESNGQPENNYKTTPPVLDSDGSFFLYSKLTVDKSRWQQGNVFSCSVMHEALHNHYT
QKSLSLSPGK

- **Fc ipilimumab (E ->D120,M->L 122) 99.05% identiy**

GPSVFLFPPKPKDTLMISRTPEVTCVVVDVSHEDPEVKFNWYVDGVEVHNAKTKPREEQYNSTYRVV
SVLTVLHQDWLNGKEYKCKVSNKALPAPIEKTISKAKGQPREPQVYTLPPSRDELTKNQVSLTCLVKG
FYPSDIAVEWESNGQPENNYKTTPPVLDSDGSFFLYSKLTVDKSRWQQGNVFSCSVMHEALHNHYT
QK SLSLSPGK

- **Fc nivolumab (H->Q32, K->Q38, Y->F60, A->G91, A->S 94,P->S119, K->R173, Q->E183, P>L209) (94.76% identity)**

GPSVFLFPPKPKDTLMISRTPEVTCVVVDVSQEDPEVQFNWYVDGVEVHNAKTKPREEQFNSYRVV
SVLTVLHQDWLNGKEYKCKVSNKGLPSSIEKTISKAKGQPREPQVYTLPPSQEEMTKNQVSLTCLVK
GFYPSDIAVEWESNGQPENNYKTTPPVLDSDGSFFLYSRLTVDKSRWQEGNVFSCSVMHEALHNHY
T QKSLSLSLGK

- **Fc pembrolizumab (H->Q32, K->Q38, Y->F60, A->G91, A->S 94,P->S119, K->R173, Q->E183, P->L209) (95.24% identity)**

GPSVFLFPPKPKDTLMISRTPEVTCVVVDVSQEDPEVQFNWYVDGVEVHNAKTKPREEQFNSTYRV
VSVLTVLHQDWLNGKEYKCKVSNKGLPSSIEKTISKAKGQPREPQVYTLPPSQEEMTKNQVSLTCLVK
GFYPSDIAVEWESNGQPENNYKTTPPVLDSDGSFFLYSRLTVDKSRWQEGNVFSCSVMHE
ALHNHYTQKS LSLSLGK

- **Fab trastuzumab**

DIQMTQSPSS LSASVGDRVT ITCRASQDVN TAVAWYQQKP GKAPKLLIYS ASFLYSGVPS RFSGSRSGTD FTLTISSLQP EDFATYYCQQ HYTTPPTFGQ GTKVEIKRTV AAPSVFIFPP SDEQLKSGTA SVVCLLNNFY PREAKVQWKV DNALQSGNSQ ESVTEQDSKD STYSLSSTLT LSKADYEKHK VYACEVTHQG LSSPVTKSFN RGECEVQLVESGGG LVQPGGSLRL SCAASGFNIK DTYIHWVRQA PGKGLEWVAR IYPTNGYTRYADSVKGRFTI SADTSKNTAY LQMNSLRAED TAVYYCSRWG GDGFYAMDYW GQGTLVTVSSASTKGPSVFP LAPSSKSTSG GTAALGCLVK DYFPEPVTVS WNSGALTSGV HTFPAVLQSSGLYSLSSVVT VPSSSLGTQT YICNVNHKPS NTKVDKKVEP KSCDKTHTCP PCPAPELLG

- **Fab NISTmAb**

DIQMTQSPSTLSASVGDRVTITCSASSRVGYMHWYQQKPGKAPKLLIYDTSKLASGVPSRFSGSGSG TEFTLTISSLQPDDFATYYCFQGSGYPFTFGGGTKVEIKRTVAAPSVFIFPPSDEQLKSGTASVVCLLN NFYPREAKVQWKVDNALQSGNSQESVTEQDSKDSTYSLSSTLTLSKADYEKHKVYACEVTHQGLSS PVTKSFNRGECQVTLRESGPALVKPTQTLTLTCTFSGFSLSTAGMSVGWIRQPPGKALEWLADIWWD DKKHYNPSLKDRLTISKDTSKNQVVLKVTNMDPADTATYYCARDMIFNFYFDVWGQGTTVTVSSAST KGPSVFPLAPSSKSTSGGTAALGCLVKDYFPEPVTVSWNSGALTSGVHTFPAVLQSSGLYSLSSVVTV PSSSLGTQTYICNVNHKPSNTKVDKRVEPKSCDKTHTCPPCPAPELLG

- **Fab ipilimumab**

EIVLTQSPGT LSLSPGERAT LSCRASQSVG SSYLAWYQQK PGQAPRLLIY GAFSRATGIP DRFSGSGSGT DFTLTISRLE PEDFAVYYCQ QYGSSPWTFG QGTKVEIKRT VAAPSVFIFP PSDEQLKSGT ASVVCLLNNF YPREAKVQWK VDNALQSGNS QESVTEQDSK DSTYSLSSTL TLSKADYEKH KVYACEVTHQ GLSSPVTKSF NRGECQVQLVESGGG VVQPGRSLRL SCAASGFTFS SYTMHWVRQA PGKGLEWVTF ISYDGNNKYYADSVKGRFTI SRDNSKNTLY LQMNSLRAED TAIYYCARTG WLGPFDYWGQ GTLVTVSSASTKGPSVFPLA PSSKSTSGGT AALGCLVKDY FPEPVTVSWN SGALTSGVHT FPAVLQSSGLYSLSSVVTVP SSSLGTQTYI CNVNHKPSNT KVDKRVEPKS CDKTHTCPPC PAPELLG

- **Fab nivolumab**

EIVLTQSPAT LSLSPGERAT LSCRASQSVS SYLAWYQQKP GQAPRLLIYD ASNRATGIPA RFSGSGSGTD FTLTISSLEP EDFAVYYCQQ SSNWPRTFGQ GTKVEIKRTV AAPSVFIFPP SDEQLKSGTA SVVCLLNNFY PREAKVQWKV DNALQSGNSQ ESVTEQDSKD STYSLSSTLT LSKADYEKHK VYACEVTHQG LSSPVTKSFN RGECQVQLVESGGG VVQPGRSLRL DCKASGITFS NSGMHWVRQA PGKGLEWVAV IWYDGSKRYYADSVKGRFTI SRDNSKNTLF LQMNSLRAED TAVYYCATND DYWGQGTLVT VSSASTKGPSVFPLAPCSRS TSESTAALGC LVKDYFPEPV TVSWNSGALT SGVHTFPAVL QSSGLYSLSSVVTVPSSSLG TKTYTCNVDH KPSNTKVDKR VESKYGPPCP PCPAPEFLG

- **Fab pembrolizumab**

EIVLTQSPAT LSLSPGERAT LSCRASKGVS TSGYSYLHWY QQKPGQAPRL LIYLASYLES GVPARFSGSG SGTDFTLTIS SLEPEDFAVY YCQHSRDLPL TFGGGTKVEI KRTVAAPSVF IFPPSDEQLK SGTASVVCLL NNFYPREAKV QWKVDNALQS GNSQESVTEQ DSKDSTYSLS STLTLSKADY EKHKVYACEV THQGLSSPVT KSFNRGECQVQLVQSGVE VKKPGASVKV SCKASGYTFT NYYMYWVRQA PGQGLEWMGG INPSNGGTNFNEKFKNRVTL TTDSSTTTAY MELKSLQFDD TAVYYCARRD YRFDMGFDYW GQGTTVTVSSASTKGPSVFP LAPCSRSTSE STAALGCLVK DYFPEPVTVS WNSGALTSGV HTFPAVLQSSGLYSLSSVVT VPSSSLGTKT YTCNVDHKPS NTKVDKRVES KYGPPCPPCP APEFLG

S-5 Separation of glycoforms of mAbs at intact level

Note that the measurements (Figures S5 and S6, and Table S5) were performed using the Ultimate RSLC3000 nano-LC system. In our experience, the performance of the Ultimate system (U3) is lower than that of the Vanquish Neo (VN) shown in the manuscript (Figure 2). Larger dead volumes (*e.g.,* valve port-to-port volumes of 20 nL VN vs 125 nL U3) and improved pump technology (allowing for better delivery of shallower gradients) result in higher performance of the separation on the VN.

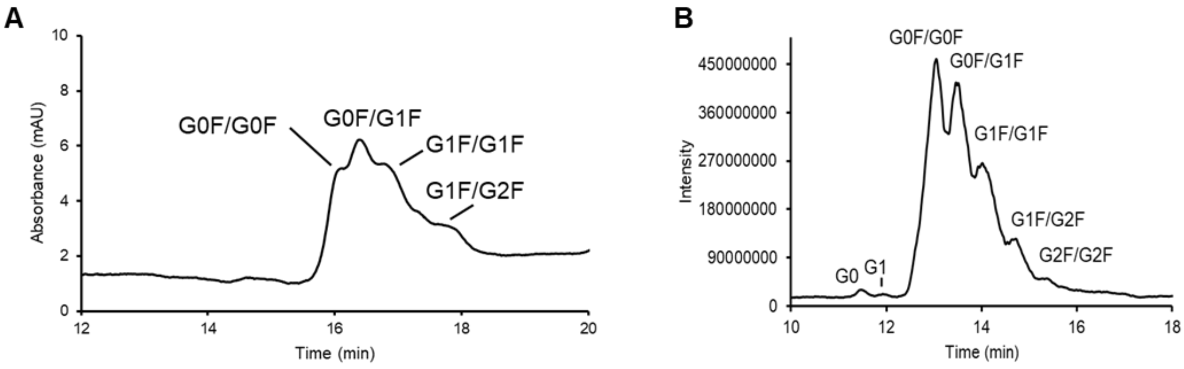

Figure S5: (A) HILIC-UV (214 nm) chromatogram of trastuzumab (5 µg injected on column) with some glycoforms tentatively annotated based on their abundance. **(B)** BPC plot of trastuzumab (100 ng injected). The glycoforms are assigned in the EICs according to Table S7.

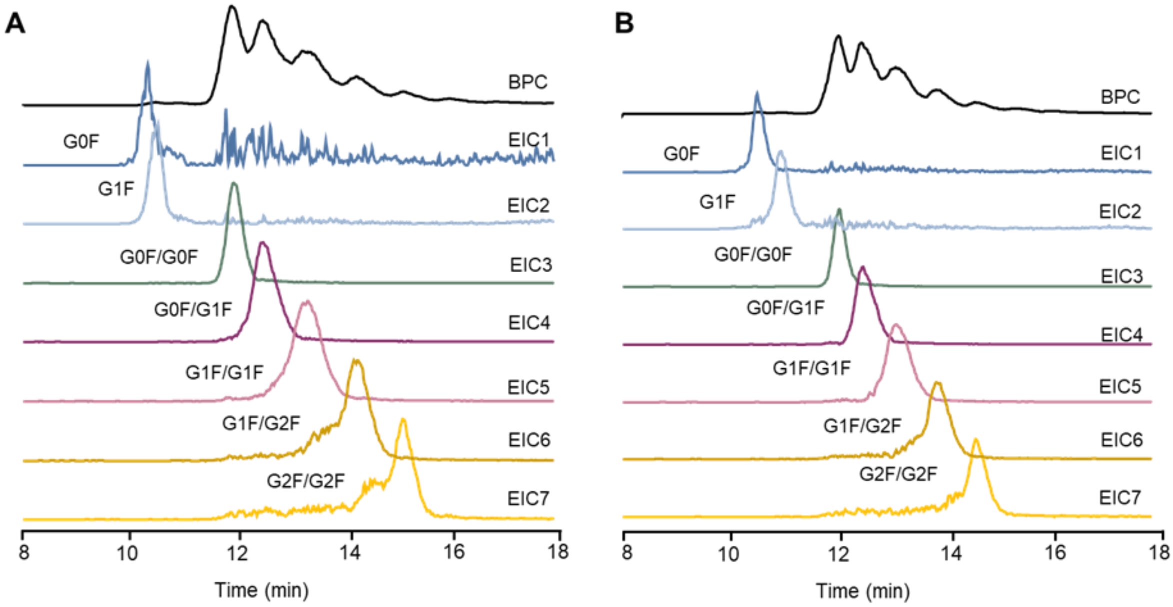

Figure S6: Comparison of performance HILIC-MS NISTmAb separation using columns **(A)** O28/D72 and **(B)** O26/D74. Isomeric structures, such as G0F/G2F, are also possible but not illustrated. The glycoforms are assigned in the EICs according to Tables S4 and S7.

Table S6: Comparison of separation performances (number of separated EIC glycoforms peaks, retention time, peak width at 50% height (width), and resolution (Rs)) of HILIC-MS analysis of NISTmAb using column conditions O28/D72 and O26/D74 (Ultimate RSLC3000 nano-LC system).

	G0F		G0F/G0F			G0F/G1F		
**mAb**	**Time[II] (min)**	**Width (min)**	**Time[II] (min)**	**Width (min)**	**Rs[III]**	**Time[II] (min)**	**Width (min)**	**Rs[IV]**
O28/D72	10.34	0.29	11.97	0.22	3.73	12.42	0.41	0.84
O26/D74	10.53	0.19	12.09	0.22	4.49	12.53	0.32	0.96

[I] EIC peaks with Rs > 0.8.; [II] 4.77 (O28/D72) min and 4.71 min (O26/D74) are subtracted from the retention time, corresponding to the time needed for the RPLC trap loading and elution to the HILIC column.; [III] Calculated between G0F and G0F/G0F.; [IV] Calculated between G0F/G0F and G0F/G1F.

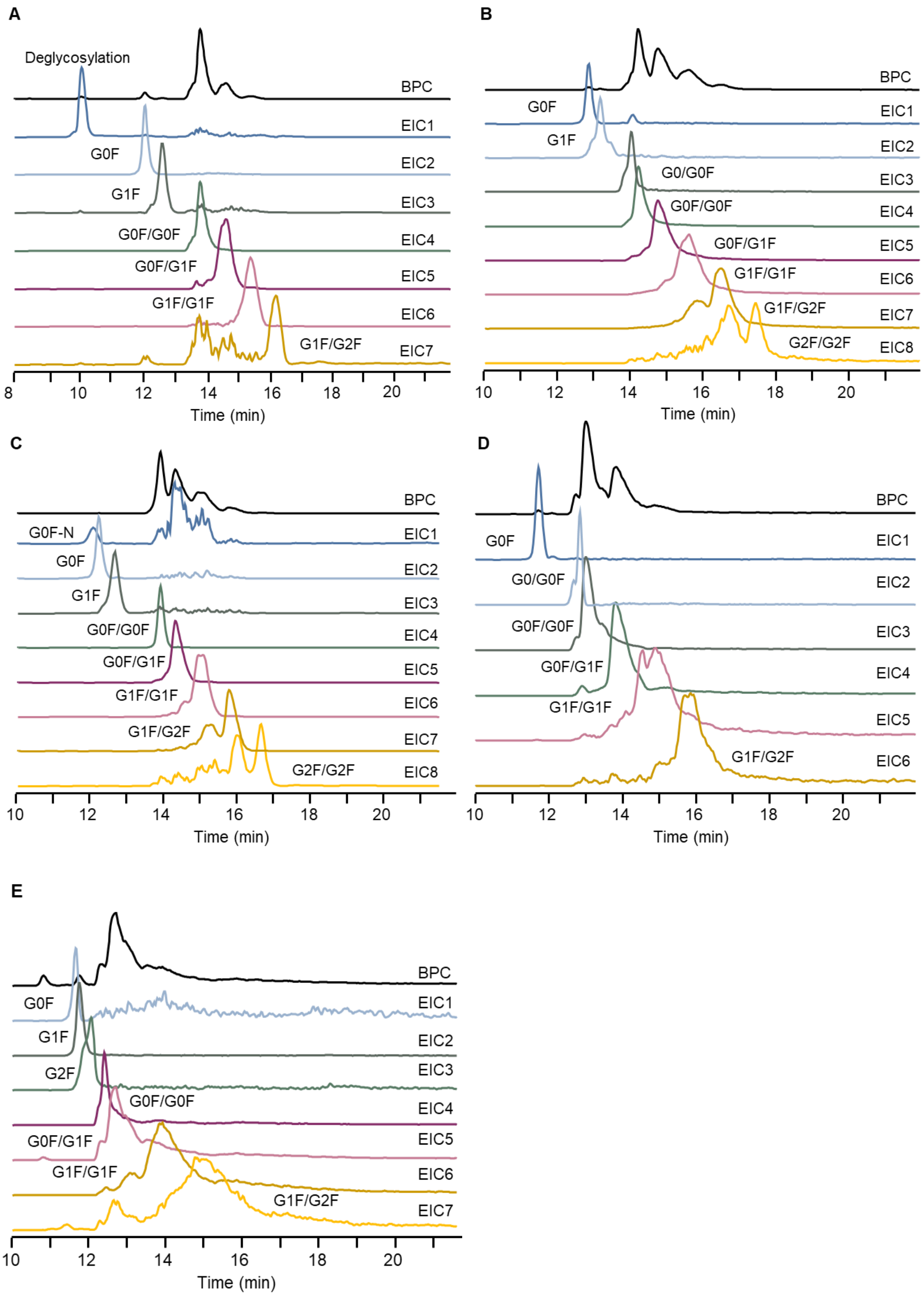

Figure S7: BPC and EIC plots of **(A)** nivolumab, **(B)** trastuzumab, **(C)** NISTmAb, **(D)** ipilimumab, and **(E)** pembrolizumab. Isomeric structures, such as G0F/G2F, are also possible but not illustrated. The glycoforms are assigned in the EICs according to Tables S4 and S7. See Experimental section for detailed LC-MS conditions.

Table S7: The *m/z* values for generating the glycoforms EICs of mAbs. A Gaussian smoothing parameter of 7 was applied with a mass tolerance of 0.50 amu.

	IgG_1-based			IgG_4-based	
**Glycoform**	**Trastuzumab (*m/z*)**	**NISTmAb (*m/z*)**	**Ipilimumab (*m/z*)**	**Pembrolizumab (*m/z*)**	**Nivolumab (*m/z*)**
Deglyco-sylation	**-**	**-**	**-**	**-**	3413.6,3496.8, 3584.1,3676.1, 3772.8
G0F-N	-	3571.7,3661.0, 3754.8,3853.6, 3957.8	-	-	-
G0F	3491.8,3597.9, 3666.4,3760.3	3576.5,3665.9, 3795.9,3858.8, 3963.2	3664.6,3758.5, 3857.4,3961.7, 4071.7	3274.3,3348.7,3426.4, 3508.2,3593.7, 3683.6	3367.9,3448.1, 3532.13,3620.4, 3713.20
G1F	3495.6,3580.9, 3670.3,3764.4, 3863.5	3580.4,3670.0, 3764.1,3863.1, 3967.6	-	3277.5,3352.1, 3430.0, 3511.7, 3597.2	3371.7,3451.9, 3536.1,3624.4, 3717.4
G2F				3281.2,3355.9, 3433.8,3515.6, 3609.3	
G0/G0F	2959.3,3019.7, 3082.5,3148.1, 3216.5	-	-	-	-
G0F/G0F	3526.2,3612.2, 3702.5,3797.4, 3897.3	3606.8,3691.9, 3786.5,3886.1, 3991.2	3610.5,3700.7, 3795.6,3895.4, 4000.7	3306.5,3381.6, 3460.2,3542.6, 3629.0	3324.2,3401.4, 3482.4,3567.4, 3656.5
G0F/G1F	3530.1,3706.6, 3801.6,3901.6	3615.7,3706.0, 3801.0,3901.0, 4006.4	3614.4,3704.8, 3799.7,3899.8, 4005.1	3309.7,3384.9, 3463.6,3546.0, 3632.5	3405.2,3486.3, 3571.3,3660.5, 3754.4
G1F/G1F	3620.2,3710.6, 3805.8,3905.9, 4011.5	3619.6,3710.1, 3805.2,3905.3, 4010.9	3618.5,3708.9, 3804.0,3904.1, 4009.6	3313.3,3467.3, 3549.9,3636.5, 3732.3	3490.1,3575.3, 3664.6,3758.6, 3857.4
G1F/G2F	3537.9,3624.1, 3714.7,3810.0, 3910.3	3623.6,3714.2, 3809.5,3909.7, 4015.3	3808.1,3908.3, 4014.0,4125.4	3392.4,3471.3, 3553.9,3640.6, 3731.6	3579.2,3668.7, 3762.8,3861.7, 3494.0
G2F/G2F	3541.7,3628.0, 3718.7,3814.0, 3914.4	3627.7,4.4,3813.4, 3813.7,3914.1, 4019.8	-	-	-

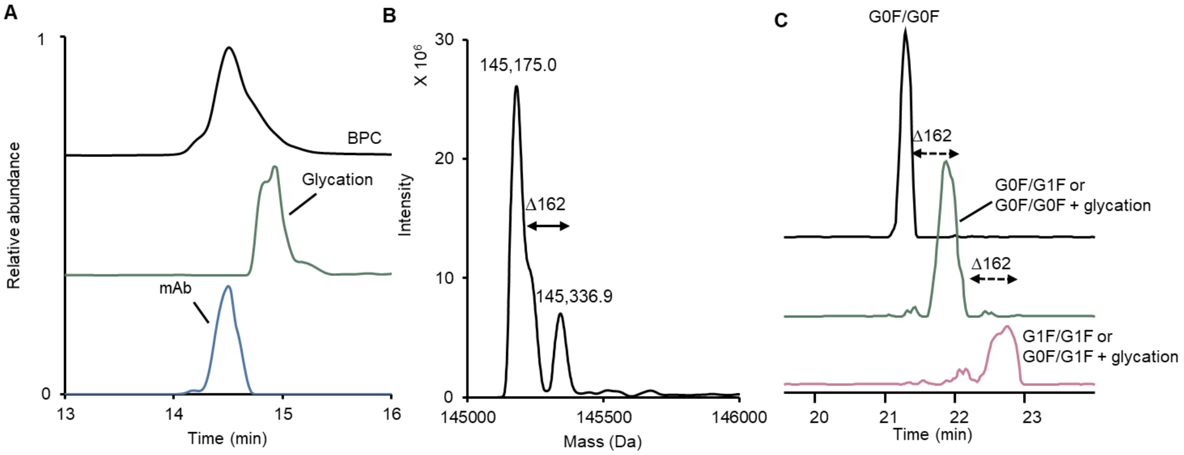

Figure S8: (A) HILIC-MS BPC and EIC plots of deglycosylated trastuzumab (blue) in which glycation (green) was assigned. **(B)** Deconvoluted spectra of deglycosylated trastuzumab. **(C)** EICs of three glycoforms (G0F/G0F, G0F/G1F, and G1F/G1F). It should be noted that an extra peak is observed for the main glycoform mass, which can indicate the presence of glycation. It should be noted that the experiments reported here were performed on a different batch of HILIC columns than the one reported in Figure S7. For the deglycosylation of trastuzumab, a solution of 0.5 mg·mL^{-1} mAb was prepared in 20 mM ammonium bicarbonate. From this, a 100 µL sample was taken and mixed with 7 µL PNGase F and left in a thermo mixer at 37 °C overnight. The deglycosylated mAb was diluted five times with water before the measurement.

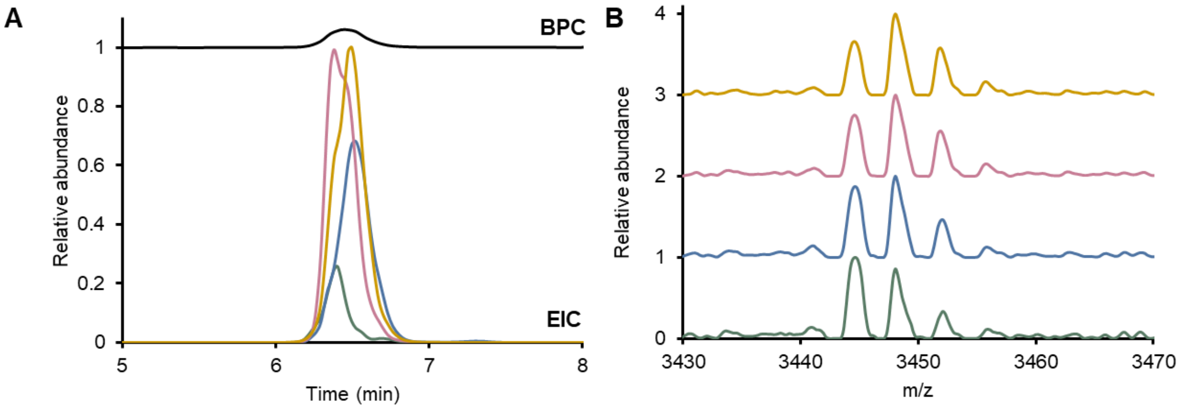

Figure S9: (A) BPC with EIC traces of glycoforms of trastuzumab (1 mg·mL^{-1}) measured with Waters ACQUITY UPLC Glycoprotein BEH Amide (300 Å, 1.7 µm, 2.1 mm x 150 mm) column. See Table S8 for detailed LC conditions. **(B)** Zoom-in *m/z* spectra of EIC traces from (A). The *m/z* values of the created EICs are listed in Table S7.

Table S8: HILIC conditions for the separation of glycoforms of trastuzumab with a commercially available column. This method was operated on an Agilent 1290 series Infinity system.

Column	Waters ACQUITY UPLC Glycoprotein BEH Amide (300 Å, 1.7 µm, 2.1 mm x 150 mm)
Mobile phase A	0.1% (v/v) TFA in water
Mobile phase B	0.1% (v/v) TFA in ACN
Flow	200 µL·min^{-1}
Column temperature	60 °C
Injection	1 µL (1 µg)
Time (min)	Mobile phase B (%)
0.00	85
0.50	85
1.00	73
21.00	71
22.00	0
23.00	0
24.00	85
25.00	85
35.00	85

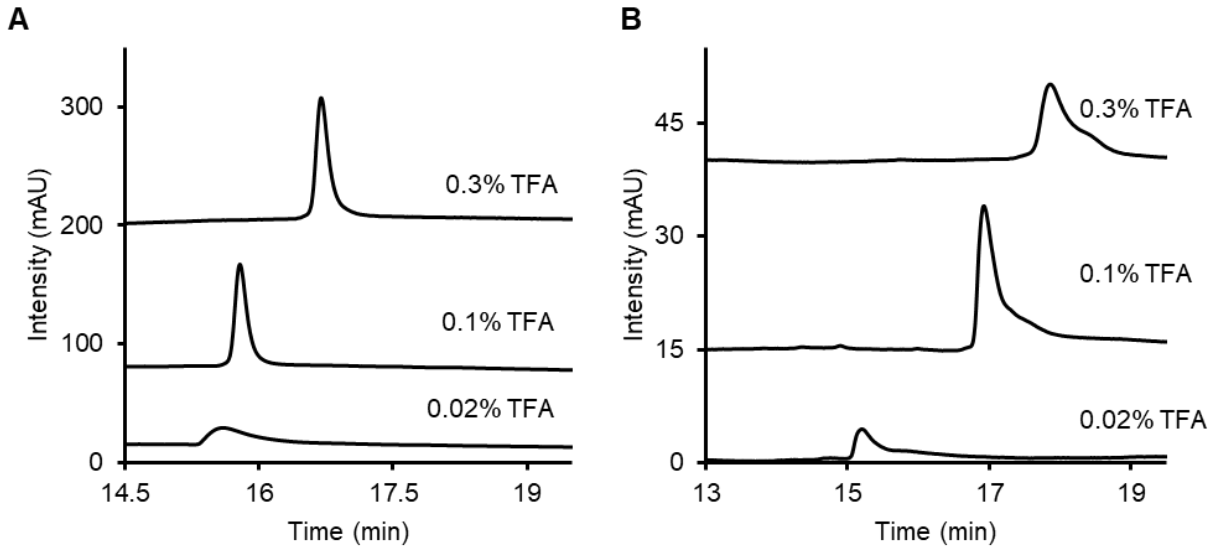

Figure S10: Influence of percentage TFA (0.02% (v/v), 0.1% (v/v), and 0.3% (v/v)) on the separation of trastuzumab using **(A)** Waters ACQUITY UPLC Glycoprotein BEH Amide (300 Å, 1.7 µm, 2.1 mm x 150 mm) column and **(B)** O26/D74 column condition. A fast gradient of 90 to 25% B was applied within 25 min. See Experimental section and Table S2 for detailed LC settings. With a higher percentage of TFA, more retention of mAb was obtained, and smaller peak widths were observed for both column measurements. However, at 0.3% (v/v) TFA (O26/D74), some separation was observed. Peak tailing was observed when a low percentage of TFA was applied for the commercial column, meaning that there are some secondary protein interactions present.

S-6 Comparison with RPLC-MS

Table S9: RPLC conditions for the separation of glycoforms of trastuzumab with a commercially available column. This method was operated on an Agilent 1290 series Infinity system.

Column	Waters ACQUITY UPLC Protein BEH C4 (1.7 µm, 300 Å, 2.1 x 50 mm)
Mobile phase A	0.1% (v/v) TFA in water
Mobile phase B	0.1% (v/v) TFA in ACN
Flow	100 µL·min^{-1}
Column temperature	60 °C
Injection	1 µL (1 µg)
Time (min)	Mobile phase B (%)
0.00	29
14.00	39
14.10	70
16.10	70
16.20	29
20.00	29

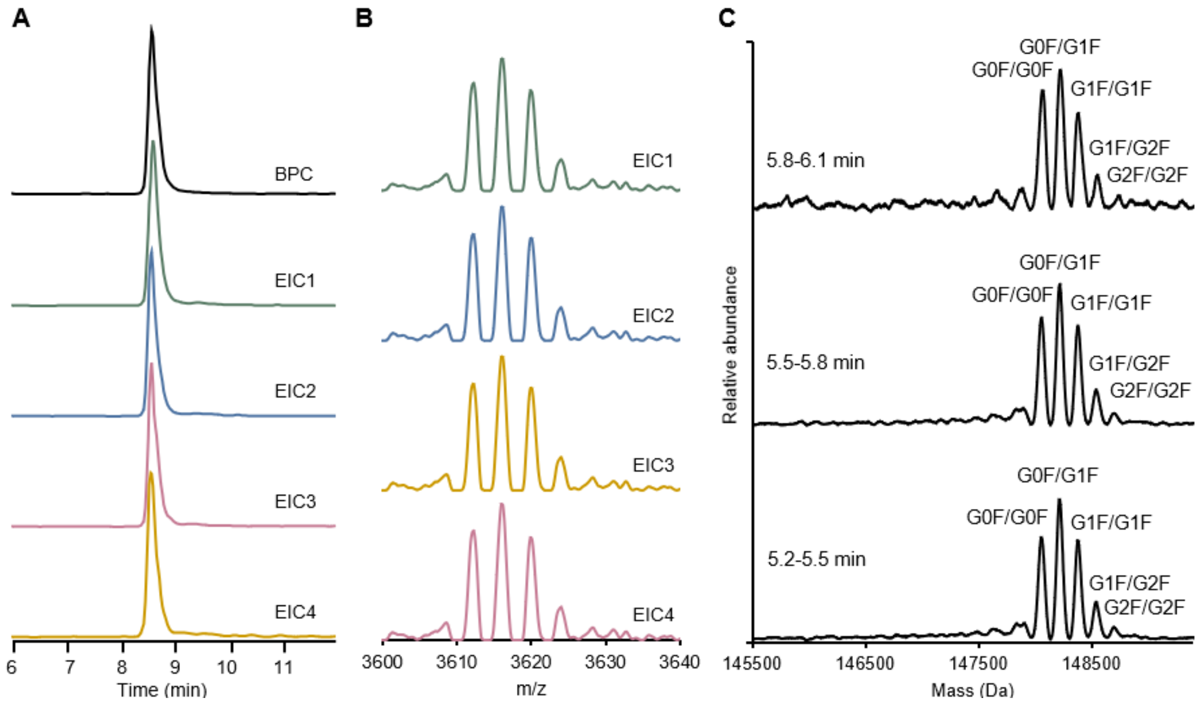

Figure S11: Trastuzumab measured with RPLC-MS **(A)** BPC and EIC plots of trastuzumab, **(B)** zoom-in *m/z* traces, and **(C)** deconvoluted spectra at different elution times. The glycoforms are annotated in the figure according to Table S4. Isomers are not included in this figure. See Table S9 for detailed separation settings, and Table S10 for additional glycoform assignments.

Table S10: Glycoform identification of trastuzumab measured with two different separation modes: HILIC and RPLC. Chromatographic conditions of RPLC and HILIC can be found in Table S9 and the Experimental section. The glycoforms are assigned according to Table S4. A mass error with a maximum of 10 Da was applied.

		HILIC			RPLC		
**Glycoforms**	**Theoretical mass (Da)**	**Mass (Da)**	**Mass error (Da)**	**D-score**	**Mass (Da)**	**Mass error (Da)**	**D-score**
G0	146,465.7	146,464.0	+1.7	74.0	-	-	-
G0F	146,611.9	146,614.3	-2.4	83.2	-	-	-
G1F	146,774.0	146,776.9	-2.9	79.8	-	-	-
G2F	146,936.2	146,941.1	-4.9	64.5	-	-	-
M5/M5	147,600.7	147,604.9	-4.2	76.8	-	-	-
M5/M6	147,762.9	147,763.0	-0.1	42.2	-	-	-
G0/G0	147,764.9	147,763.0	+1.9	42.2	-	-	-
G0/G0F	147,911.1	147,914.6	-3.5	59.9	-	-	-
G0F/G0F	148,057.2	148,060.5	-2.8	79.6	148,057.6	-0.6	69.2
G0F/G1F	148,219.3	148,222.1	-3.2	80.5	148,217.7	+1.6	73.8
G1F/G1F	148,381.5	148,384.5	-3.0	78.7	148,381.7	-0.2	66.9
G0F/G2F	148,381.5	148,384.5	-3.0	78.7	148,381.7	-0.2	66.9
G1F/G2F	148,543.7	148,549.1	-5.4	68.9	148,539.7	+4.0	50.6
G2F/G2F	148,705.8	148,711.2	-5.4	68.8	148,697.8	+8.0	38.3
G2F/G2F+ 1 mannose	148,867.8	148,861.8	+5.7	65.8	-	-	-
G2F/G3F	148,867.8	148,861.8	+5.7	65.8	-	-	-
G2F/G2S1	148,997.1	149,006.5	-9.4	59.2	-	-	-

References

[1] Courtois J, Szumski M, Byström E, Iwasiewicz A, Shchukarev A, Irgum K. A study of surface modification and anchoring techniques used in the preparation of monolithic microcolumns in fused silica capillaries. J Sep Sci 2006;29:325–325. https://doi.org/10.1002/jssc.200690007.

[2] Neelamegham S, Aoki-Kinoshita K, Bolton E, Frank M, Lisacek F, Lütteke T, et al. Updates to the Symbol Nomenclature for Glycans guidelines. Glycobiology 2019;29:620–4. https://doi.org/10.1093/glycob/cwz045.

[3] Goyon A, Excoffier M, Janin-Bussat M-C, Bobaly B, Fekete S, Guillarme D, et al. Determination of isoelectric points and relative charge variants of 23 therapeutic monoclonal antibodies. Journal of Chromatography B 2017;1065–1066:119–28. https://doi.org/10.1016/j.jchromb.2017.09.033.

[4] Turner A, Schiel JE. Qualification of NISTmAb charge heterogeneity control assays. Anal Bioanal Chem 2018;410:2079–93. https://doi.org/10.1007/s00216-017-0816-6.

[5] Lippold S, Nicolardi S, Wuhrer M, Falck D. Proteoform-Resolved FcɣRIIIa Binding Assay for Fab Glycosylated Monoclonal Antibodies Achieved by Affinity Chromatography Mass Spectrometry of Fc Moieties. Front Chem 2019;7. https://doi.org/10.3389/fchem.2019.00698.

[6] Füssl F, Trappe A, Carillo S, Jakes C, Bones J. Comparative Elucidation of Cetuximab Heterogeneity on the Intact Protein Level by Cation Exchange Chromatography and Capillary Electrophoresis Coupled to Mass Spectrometry. Anal Chem 2020;92:5431–8. https://doi.org/10.1021/acs.analchem.0c00185.
